# Contribution to the Knowledge of Dicranoptychini (Diptera, Tipuloidea, Limoniidae) in China, with the First Mitochondrial Genome of the Tribe and Its Phylogenetic Implications

**DOI:** 10.3390/insects14060535

**Published:** 2023-06-07

**Authors:** Yuanyuan Xu, Shenglin Zhang, Yaru Chen, Guoquan Wang, Ding Yang, Xiao Zhang

**Affiliations:** 1 Guangxi Key Laboratory of Agric-Environment and Agric-Products Safety, National Demonstration Center for Experimental Plant Science Education, Agricultural College, Guangxi University, Nanning 530004, China; 2 Shandong Engineering Research Center for Environment-Friendly Agricultural Pest Management, College of Plant Health and Medicine, Qingdao Agricultural University, Qingdao 266109, China; 3 Department of Entomology, China Agricultural University, Beijing 100193, China

**Keywords:** taxonomy, phylogeny, Chinese fauna, new species, new record, identification key, mitogenome, Dicranoptychinae, *Dicranoptycha*

## Abstract

**Simple Summary:**

Limoniidae, the most speciose family in the superfamily Tipuloidea (crane flies), consists of four subfamilies (i.e., Chioneinae, Dactylolabidinae, Limnophilinae, and Limoniinae) and more than 11,000 species. However, its four-subfamily classification system has been controversial, among which the taxonomic position of the tribe Dicranoptychini (Limoniinae) is subject to debate. In addition, the knowledge of species diversity and geographical distribution of the tribe in China is seriously insufficient. We conducted investigations on Dicranoptychini in China and examined specimens from several Chinese localities to discover new species and new records and selected suitable materials for mitochondrial (mt) genome sequencing. In this study, two new and one newly recorded species from China have been found, which increases the number of Dicranoptychini species in the Chinese Mainland from four to seven, and adds three new provincial distribution records for the tribe in China. The first key for all Chinese *Dicranoptycha* species based on the type and non-type specimens and literature is also presented. The mt genome of one of the new species is sequenced and annotated, representing the first mt genome of the tribe Dicranoptychini. Phylogenetic analysis supports both new and traditional arrangements. This study will improve the knowledge of Chinese Dicranoptychini and provide genomics data and new horizons for the phylogeny of Limoniidae.

**Abstract:**

Dicranoptychini is a tribe in the subfamily Limoniinae (Diptera, Tipuloidea, and Limoniidae) and includes only the genus *Dicranoptycha* Osten Sacken, 1860. However, the species diversity of the tribe in China was seriously underestimated, and the taxonomic status of *Dicranoptycha* has long been controversial. In this study, types of Chinese *Dicranoptycha* species and specimens collected from several localities in China were examined, and the first mitochondrial (mt) genome of the tribe Dicranoptychini is presented. Two *Dicranoptycha* species, *D. jiufengshana* sp. nov. and *D. shandongensis* sp. nov., from China, are described and illustrated as new to science. A Palaearctic species, *D. prolongata* Alexander, 1938, is recorded in China for the first time. In addition, the complete mt genome of *D. shandongensis* sp. nov. is sequenced and annotated, indicating that it is a typical circular DNA molecule with a length of 16,157 bp and shows a similar gene order, nucleotide composition, and codon usage to mt genomes of other Tipuloidea species. The two pairs of repeat elements are found in its control region. Phylogenetic results confirm the sister-group relationship between Cylindrotomidae and Tipulidae, question the position of the genus *Epiphragma* Osten Sacken, 1860 in Limoniidae, and indicate that Dicranoptychini may be a basal lineage within Limoniinae.

## 1. Introduction

With more than 11,000 species in about 150 genera, the family Limoniidae (Diptera, Tipuloidea) is one of the species-richest groups of flies and is further subdivided into four subfamilies: Chioneinae/Eriopterinae, Dactylolabidinae, Limnophilinae/Hexatominae and Limoniinae [1,2]. However, the delineations of these subfamilies are controversial [3]. Within Tipuloidea, Pediciidae is sister to all remaining Tipuloidea (i.e., Limoniidae, Tipulidae, and Cylindrotomidae) as the earliest lineage, which was widely supported by Ribeiro (2008) [4], Petersen et al. (2010) [3], Zhang et al. (2016) [5] and Kang et al. (2017) [6]. However, several studies based on morphological and molecular data indicated that Limoniidae is a paraphyletic group and the origin of Tipulidae and Cylindrotomidae comes from a part of Limoniidae [3,4,5].

Dicranoptychini is one of the tribes in the subfamily Limoniinae and includes only the genus *Dicranoptycha* Osten Sacken, 1860 [7,8]. However, the taxonomic position of the genus *Dicranoptycha* has been subject to debate. Based on the reduced meron, the absence of tibial spurs and the absence of cell m_1_, Savchenko (1982) transferred *Dicranoptycha* to Gonomyini (Chioneinae) but considered this genus as an “aberrant representative of Eriopterinae” [9]. In a study by Savchenko (1989), *Dicranoptycha* was also listed among Gonomyini [10]. Later, Starý (1992) transferred *Dicranoptycha* to Limoniinae because of the two-branched radial sector of the wing, which is common in Limoniinae [1]. In most Chioneinae, the radial sector of the wing branches into two veins. The phylogeny of Tipuloidea based on characters of larvae and pupae indicated that *Dicranoptycha* strongly diverged from Limoniinae and Chioneinae [11], which was supported by Petersen et al. (2010) based on the combined morphological characters (adult, larvae, and pupae) and nuclear gene sequence data (28S rDNA and CAD) [3]. The recovery of *Dicranoptycha* as an independent lineage is similar to the placement of *Dactylolabis* Osten Sacken, 1860, so Petersen et al. (2010) proposed to treat *Dicranoptycha* as a separate subfamily, Dicranoptychinae, and defined this subfamily through a combination of characters, including completeness of larval head capsule, dorsal larval creeping welts absent, dorsal and ventral pupal creeping welts, wing venation, tibial spurs, antennal flagellomeres and male genetalic characters [3]. However, in a study by Starý (2021), *Dicranoptycha* was treated as a basal lineage within the Limoniinae [12].

*Dicranoptycha* is a cosmopolitan genus with 87 extant species from the Afrotropic (31 species), Palaearctic (24 species), Nearctic (23 species), Oriental (seven species), and Neotropic (three species) regions [2]. Nine fossil *Dicranoptycha* species have been found so far, among which the oldest representatives are from the Upper Cretaceous Burmese amber [13]. The most distinctive feature of the genus is the wing with a fold in the cell cu, which is not found in other genera of Limoniidae. The members of *Dicranoptycha* usually have one generation a year. Larvae are often found in relatively dry soil, such as dry woodland traversed by a small stream that is entirely dry [14], and open woodland far from running water [15]. Adults are usually found in open woodlands, often but not necessarily near water [14,15,16,17,18]. They often rest on the upper surface of the leaves of tall herbs and low shrubs [14], or in the undergrowth stratum of shrubs and herbs [18]. In addition, there are also some specialized dry-psammophilous species [19,20].

In the last half-century, taxonomic studies on the genus *Dicranoptycha* in the world have mainly focused on European and American species. A large number of studies have been carried out in Europe [17,19,20,21,22,23]. Chen Wen Young made important contributions to the taxonomy of *Dicranoptycha* in North America [18]. Some taxonomic studies on *Dicranoptycha* in Asia have only been carried out in the past decade, mainly focusing on species of the Korean peninsula [24,25,26]. These studies have increased the number of *Dicranoptycha* species in the Korean peninsula from two to five. At present, Japan has the richest fauna of *Dicranoptycha* crane flies in Asia, with a total of nine species [2]. Only four *Dicranoptycha* species are known to occur in the Chinese Mainland, which spans the Palaearctic and Oriental regions: *D. kwangtungensis* Alexander, 1942, from Guangdong; *D. pallidibasis* Alexander, 1931, from Hebei; *D. phallosomica* Alexander, 1937, from Ningxia, Zhejiang, and Jiangxi; and *D. vulpes* Alexander, 1935, from Sichuan [2]. No new *Dicranoptycha* species from the Chinese Mainland have been described for more than 80 years, and the lack of systematic investigation for a long time has led to a serious underestimation of the species diversity of the genus.

To improve the understanding of the species diversity of *Dicranoptycha* crane flies in China, some investigations were initiated by the authors together with other entomologists. We examined specimens from several localities in China, resulting in the discovery of three *Dicranoptycha* species, of which *D. jiufengshana* sp. nov. from Inner Mongolia and *D. shandongensis* sp. nov. from Shandong are described as new to science, and *D. prolongata* Alexander, 1938, previously known from the Far East of Russia, North Korea, and South Korea, is recorded in China for the first time. The descriptions and illustrations of the males and females, as well as the first key to Chinese *Dicranoptycha* crane flies based on the type and non-type specimens and literature, are presented. Due to maternal inheritance, rapid evolution, and highly conserved gene content, the mitochondrial (mt) genome has been widely used in insect phylogenetic research [27,28,29,30,31,32,33,34,35,36,37,38,39,40,41,42,43,44,45,46,47,48,49,50,51,52,53,54]. In 2016, we reported the first three mt genomes of Limoniidae [5]. After that, we and other researchers have successively published some mt genomes of Limoniidae [5,55,56,57,58,59]. Prior to this study, 11 complete or nearly complete mt genomes of Limoniidae have been included in the GenBank. However, mt genomes that can be used for phylogenetic study are far from sufficient. To provide genomics data and new horizons for the phylogeny of Limoniidae, we sequenced and annotated the complete mt genome of *D. shandongensis* sp. nov., representing the first mt genome of the tribe Dicranoptychini. Based on this new sequence and previously published sequences, the phylogenetic relationships of Limonidae are also reconstructed. The monophyly of Limoniidae and the taxonomic position of Dicranoptychini will be mainly explored.

## 2. Materials and Methods

### 2.1. Specimen Collection, Observation, and Description

Specimens for this study were collected from several localities in China by different entomologists from 2013 to 2022. The type specimens of all Chinese *Dicranoptycha* species, deposited in the National Museum of Natural History, Smithsonian Institution, Washington, DC, USA (USNM) and the Institute of Zoology, Chinese Academy of Sciences, Beijing, China (IOZ), were examined during the study (Table 1). The type specimens of *D. jiufengshana* sp. nov. are deposited in the Entomological Museum of China Agricultural University, Beijing, China (CAU). Other specimens including the type specimens of *D. shandongensis* sp. nov. are deposited in the Entomological Museum of Qingdao Agricultural University, Shandong, China (QAU). Genitalic preparations were made by macerating the apical portion of the abdomen in cold 10% sodium hydroxide for 12–15 h. The ZEISS Stemi 2000-C stereomicroscope was used for observations and illustrations. The Canon EOS 90D digital camera was used to take photos through a macro lens. The terminal abdomens were macerated in cold 10% sodium hydroxide for 12–15 h to better observe the genitalic structure. The specimens were soaked in 75% ethanol when examining the details of coloration. The morphological terminology mainly followed Cumming and Wood (2017) [60], while the terminologies for venation and terminalia followed de Jong (2017) [61] and Young (1987) [18], respectively. The following abbreviations in figures were used: aa = anterior apodeme; abc = aperture of bursa copulatrix; aed = aedeagus; aep = aedeagal process; cerc = cercus; gf = genital furca; goncx = gonocoxite; hyp vlv = hypogynial valve; i gonst = inner gonostylus; lp = lateral process; o gonst = outer gonostylus; st = sternite; tg = tergite; ve = vesica.

### 2.2. Sampling, DNA Extraction, and Sequencing

A male non-type specimen of *D. shandongensis* sp. nov. collected by Yuanyuan Xu on 23 June 2021, from Zhongshan Temple in Mengyin County, Shandong Province, China (35°47′54″ N, 118°10′8″ E, 306 m) was used for DNA extraction. The specimen was preserved in 96% ethanol at −20 °C for long-term storage at QAU. The genomic DNA was extracted from the muscle tissue of the thorax using the TIANamp Genomic DNA Kit (TIANGEN) according to the manufacturer’s protocol. The whole genome was sequenced with 150 bp paired-end reads on the Illumina Hiseq 2500 platform at Biomarker Technologies (Qingdao, Shandong, China), and about 20 Gb of clean data were obtained.

### 2.3. Mitochondrial Genome Assembly, Annotation, and Analysis

Assembly and annotation were conducted using MitoZ 3.5 (https://github.com/linzhi2013/MitoZ/wiki/Citations, accessed on 25 March 2023) [62] and then checked by manual proofreading according to its relative species. The gene map was drawn with OGDRAW (https://chlorobox.mpimp-golm.mpg.de/OGDraw.html, accessed on 3 April 2023). Nucleotide composition and codon usage were analyzed with MEGA 7.0 [63]. The AT skew [(A − T)/(A + T)] and GC skew [(A − T)/(G + C)] were calculated manually [64]. The tandem repeats in the AT-rich region were identified using the Tandem Repeats Finder server (http://tandem.bu.edu/trf/trf.html, accessed on 10 April 2023).

### 2.4. Phylogenetic Analysis

A total of 18 mt genomes were used for molecular analysis, including the newly sequenced mt genome in this study, 11 published mt genomes, and 6 mt genomes provided by the DNAmark Project conducted by F. Leerhoei in NCBI (https://www.ncbi.nlm.nih.gov/, accessed on 11 April 2023) (Table 2). All complete or nearly complete mt genomes of Limoniidae available in the GenBank of NCBI were used. Some mt genomes of Cylindrotomidae and Tipulidae were also selected to verify the monophyly of Limoniidae. The mitochondrial genome of Pediciidae was used as an outgroup.

Individual gene alignments were checked manually in MEGA 7.0. Alignments of individual genes were concatenated using SequenceMatrix 1.8 [65] to generate the following four datasets: (1) PCGRNA matrix, including all three codon positions of 13 protein-coding genes (PCGs), and all available transfer RNA (tRNA) and ribosomal RNA (rRNA) genes (11,353 bp); (2) PCG matrix, including all three codon positions of 13 PCGs (9444 bp); (3) PCG12RNA matrix, including the first and second codon positions of 13 PCGs, and all available transfer RNA (tRNA) and ribosomal RNA (rRNA) genes (8205 bp); (4) PCG12 matrix, including the first and second codon positions of 13 PCGs (6296 bp).

Phylogenetic trees inferred from the four datasets were constructed under Bayesian inference (BI) and maximum likelihood (ML) methods. MrBayes 3.2.7 [66] and RAxML 8.2.4 [67] were used for BI and ML analyses, respectively. The BIC criterion and greedy search algorithm in PartitionFinder 2.1.1 [68] were used to determine partitioning schemes and the best-fitting substitution models. In the BI analysis, four runs of 5–10 million generations were conducted simultaneously until the average standard deviation of split frequencies was below 0.01; samples were taken every 1000 generations and the initial 25% generations were discarded as burn-in. In the ML analysis, the GTRGAMMAI model was used, and the best ML tree was calculated with branch support estimated at 1000 bootstrap replicates.

**Table 2:** Information of species used in phylogenetic analysis with the GenBank accession numbers of mitochondrial genome sequences.

Family/Subfamily	Species	Accession Number	Reference
Pediciidae	*Pedicia* sp.	KT970062	Zhang et al. (2016) [5]
Chioneinae	*Chionea crassipes gracilistyla* Alexander, 1936	MK941181	Kang et al. (2019) [55]
	*Symplecta* (*Symplecta*) *hybrida* (Meigen, 1804)	NC_030519	Zhang et al. (2016) [5]
Limnophilinae	*Conosia irrorata* (Wiedemann, 1828)	NC_057072	Zhang et al. (2019) [58]
	*Epiphragma* (*Epiphragma*) *mediale* Mao and Yang, 2009	NC_057085	Zhang et al. (2021) [59]
	*Euphylidorea* (*Euphylidorea*) *dispar* (Meigen, 1818)	MT410841	/
	*Paradelphomyia* sp.	KT970061	Zhang et al. (2016) [5]
	*Pseudolimnophila* (*Pseudolimnophila*) *brunneinota* Alexander, 1933	MN398932	Ren et al. (2019) [57]
Limoniinae	*Dicranomyia* (*Dicranomyia*) *modesta* (Meigen, 1818)	MT628560	/
	*Dicranoptycha shandongensis* sp. nov.	OR074105	This study
	*Limonia phragmitidis* (Schrank, 1781)	NC_044484	Ren et al. (2019) [56]
	*Metalimnobia* (*Metalimnobia*) *quadrinotata* (Meigen, 1818)	MT584154	/
	*Rhipidia* (*Rhipidia*) *chenwenyoungi* Zhang, Li and Yang, 2012	KT970063	Zhang et al. (2016) [5]
Cylindrotomidae	*Cylindrotoma* sp.	KT970060	Zhang et al. (2016) [5]
Tipulidae	*Tanyptera* (*Tanyptera*) *hebeiensis* Yang and Yang, 1988	NC_053795	Zhao et al. (2021) [69]
	*Nephrotoma flavescens* (Linnaeus, 1758)	MT628586	/
	*Nigrotipula nigra nigra* (Linnaeus, 1758)	MT483653	/
	*Tipula* (*Tipula*) *paludosa* Meigen, 1830	MT483696	/

## 3. Results and Discussion

### 3.1. Taxonomy

#### 3.1.1. *Dicranoptycha jiufengshana* Xu, Chen and Zhang, sp. nov.

Diagnosis. Antenna with scape brownish yellow; pedicel yellow; first flagellomere brownish yellow with brown tip, remaining flagellomeres brown. Setae on flagellomeres just slightly exceeding the length of respective segments. Prescutum and presutural scutum brownish black. Pleuron dark brown. Wing with stigma indistinct, not darker than remaining wing area; Rs slightly longer than cell dm; cell dm about three times as long as wide; m-cu about its length beyond fork of M and approximately at two-fifth of cell dm. Outer gonostylus with smooth lower and upper margins. Aedeagal process umbrella-shaped, connected beneath the margin to a rod-like lower part. Aedeagus short and broad, concave at the tip, with two hook-like sclerites at the base. Aperture of bursa copulatrix in females with two sharp corners.

Description. Male. Body length 12.0 mm, wing length 10.5 mm.

**Figure 1:**
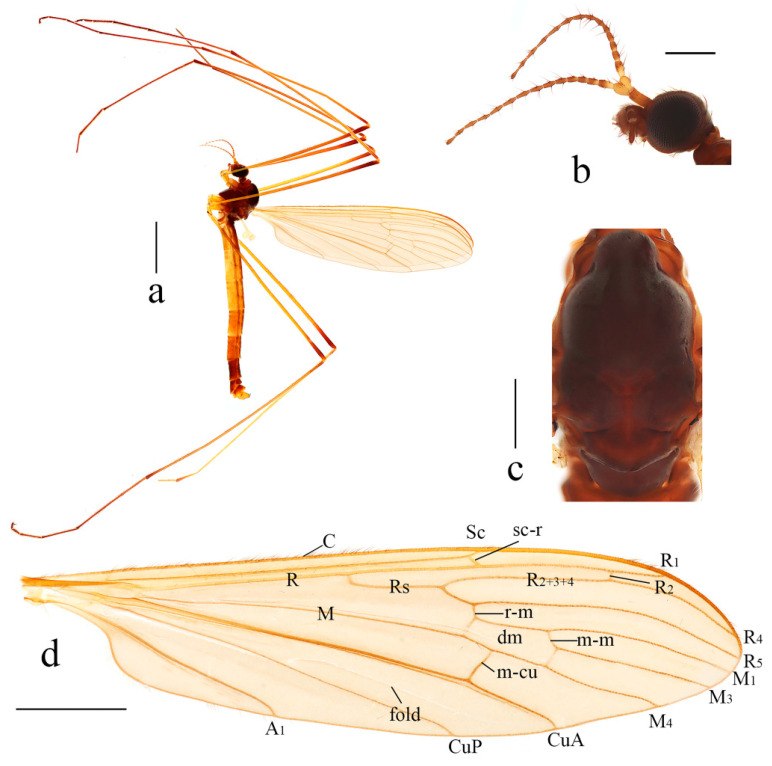
*Dicranoptycha jiufengshana* sp. Nov. (**a**) Habitus of male, lateral view; (**b**) head, lateral view; (**c**) thorax, dorsal view; (**d**) wing. Scale bars: 2.0 mm (**a**); 0.5 mm (**b**,**c**); 1.5 mm (**d**).

Head (Figure 1b), dark brown. Hairs on head dark brown. Antenna length 1.9 mm; scape brownish yellow; pedicel yellow; first flagellomere brownish yellow with brown tip, remaining flagellomeres brown. Scape long and cylindrical, 2.5 times as long as wide; pedicel oval, widened distally; flagellomeres subcylindrical, tapering apically and elongated. Setae on flagellomeres just slightly exceeding the length of respective segments. Mouthparts dark brown with dark brown hairs; palpus dark brown with dark brown hairs.

Thorax (Figure 1c). Pronotum dark brown. Prescutum and presutural scutum uniformly brownish black. Postsutural scutum brownish black, paler medially and laterally. Scutellum brownish black, slightly paler medially. Mediotergite brownish black. Pleuron (Figure 1a) dark brown, without stripes. Hairs on thorax white. Coxae and trochanters yellow; femora brownish yellow with wide dark brown tip; tibiae brownish yellow with wide brownish black tip; tarsi dark brown. Hairs on legs dark brown. Wing (Figure 1d) uniformly tinged with brownish yellow, except yellow costal field. Stigma indistinct, not darker than the remaining wing area. Veins brown, CuA and veins at wing base and in costal area brownish yellow. Venation: Sc long, ending conspicuously beyond fork of Rs; sc-r close to the tip of Sc; Rs slightly longer than cell dm; cell dm elongated, about three times as long as wide; m-cu about its length beyond fork of M, approximately at two-fifth of cell dm. Halter white, its length 1.5 mm.

**Figure 2:**
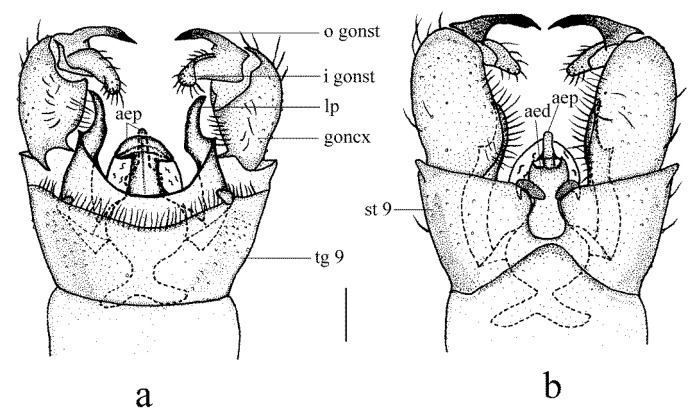
*Dicranoptycha jiufengshana* sp. Nov. (**a**) Male hypopygium, dorsal view; (**b**) male hypopygium, ventral view. Scale bar: 0.2 mm.

**Figure 3:**
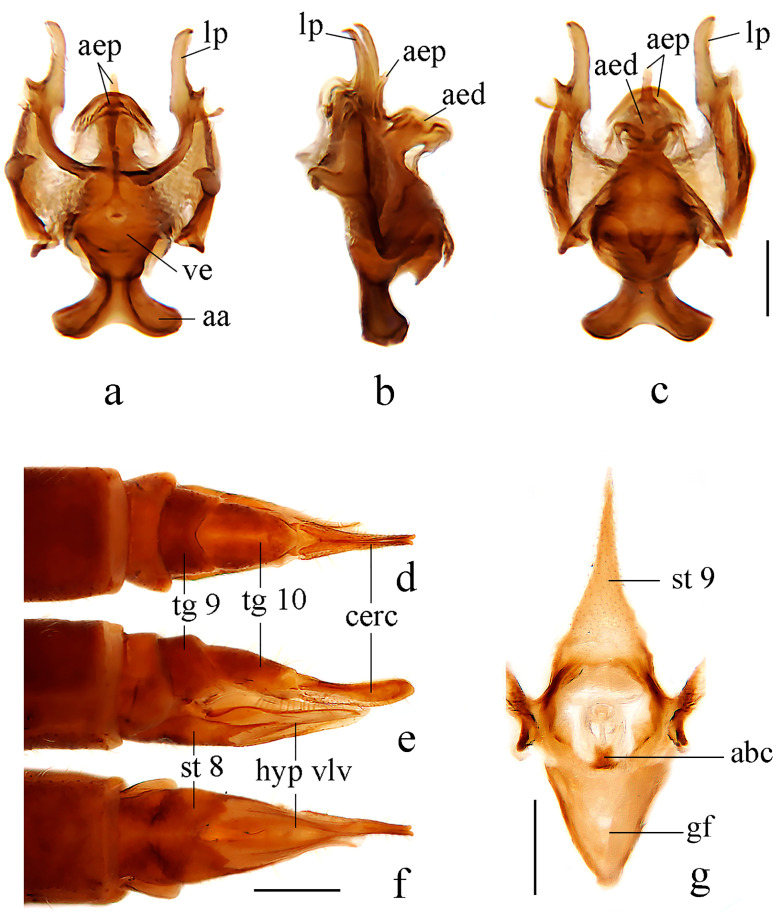
*Dicranoptycha jiufengshana* sp. Nov. (**a**) Complex of aedeagus, dorsal view; (**b**) complex of aedeagus, lateral view; (**c**) complex of aedeagus, ventral view; (**d**) female ovipositor, dorsal view; (**e**) female ovipositor, lateral view; (**f**) female ovipositor, ventral view; (**g**) vaginal apodeme. Scale bars: 0.2 mm (**a**–**c**,**g**); 0.5 mm (**d**–**f**).

Hypopygium (Figure 2 and Figure 3a–c). Posterior margin of tergite 9 with a broad and shallow V-shaped emargination. Posterior margin of sternite 9 with a U-shaped emargination. Gonocoxite simple, elongate-oval. Outer gonostylus sclerotized distally and curved inwards with pointed apex, lower and upper margins smooth. Inner gonostylus nearly as long as the outer gonostylus, wide and fleshy, curved at two-thirds of its length, with a blunt apex and covered with sparse setae. Lateral process curved outwards, wide at the base and narrowed distally, apex pointed (Figure 2). Anterior apodemes slender at the point of fusion with vesica, widening to broadly rounded apices. Aedeagal process umbrella-shaped, connected beneath the margin to a rod-like lower part. Aedeagus short and broad, concave at the tip, with two hook-like sclerites at the base (Figure 2 and Figure 3a–c).

Female. Body length 11.5 mm, wing length 10.0 mm. Similar to male, but abdomen with segments 1–8 dark brown. Tergite 9 brownish yellow, posterior margin with a broad V-shaped emargination. Tergite 10 brownish yellow, base, and middle area paler. Cercus brownish yellow, caudal half widened and apex blunt. Hypogynial valve reaching approximately two-thirds of the cercus, setae on the dorsal margin long and distinct (Figure 3d–f). Vaginal apodeme as shown in Figure 3g, aperture of bursa copulatrix with two sharp corners.

Type material. Holotype: male, China: Inner Mongolia Autonomous Region, Tumd Right Banner, Mount Jiufengshan, Toudaogou, 23 August 2013, Xiao Zhang. Paratype: 1 female, same data as holotype.

Distribution. China (Inner Mongolia).

Etymology. The species name *jiufengshana* (adjective, feminine) refers to the type locality, Mount Jiufengshan.

Remarks. This species is somewhat similar to *D. buksubaeksaniana* Podenas, Byun, and Kim, 2015, from North and South Korea with a similar male hypopygium (see Figure 11 in Podenas et al. 2015 [25]) but can be easily distinguished from the latter by the antenna with a pedicel lighter rather than the scape (Figure 1b), the uniform brownish black prescutum and presutural scutum (Figure 1c), the femora and the tibiae with wide darker tips (Figure 1a), and the vein Sc ending conspicuously beyond the fork of Rs (beyond the crossvein r-m). In *D. buksubaeksaniana*, the pedicel of the antenna is darker than the scape, the prescutum and presutural scutum have four dark brown longitudinal stripes, the femora, and tibiae have narrowly darkened distal ends [25], and the vein Sc ends slightly beyond the fork of Rs (before the crossvein r-m) (see Figure 10 in Podenas et al. 2015 [25]). The discovery of this species indicates that the tribe Dicranoptychini is recorded in Inner Mongolia, China, for the first time.

#### 3.1.2. *Dicranoptycha prolongata* Alexander, 1938

*Dicranoptycha prolongata* Alexander, 1938: 139 [70]. Type locality: Ompo (North Korea).

Diagnosis. Antenna with scape and pedicel dark yellow; first flagellomere dark yellow at base, remaining flagellomeres brown. Setae on flagellomeres just slightly exceeding the length of respective segments. Brown prescutum and presutural scutum with three broad dark brown longitudinal stripes, median stripe subdivided by a narrow line of brownish black. Pleuron dark brown with indistinct black longitudinal stripe. Wing wide; stigma indistinct, not darker than remaining wing area. Rs subequal to or slightly longer than cell dm Rs and dm; m-cu about its length beyond fork of M and approximately at two-fifth of cell dm. Outer gonostylus with outer and inner margins of the tip serrated. Aedeagal process with flattened upper part emarginates at the posterior margin, hook-like lower part bent downward across aedeagus. Aedeagus with a U-shaped emargination, each side with the apex pointed medially. Aperture of bursa copulatrix in females oval.

Description. Male. Body length 9.0–9.4 mm, wing length 9.5–10.0 mm.

**Figure 4:**
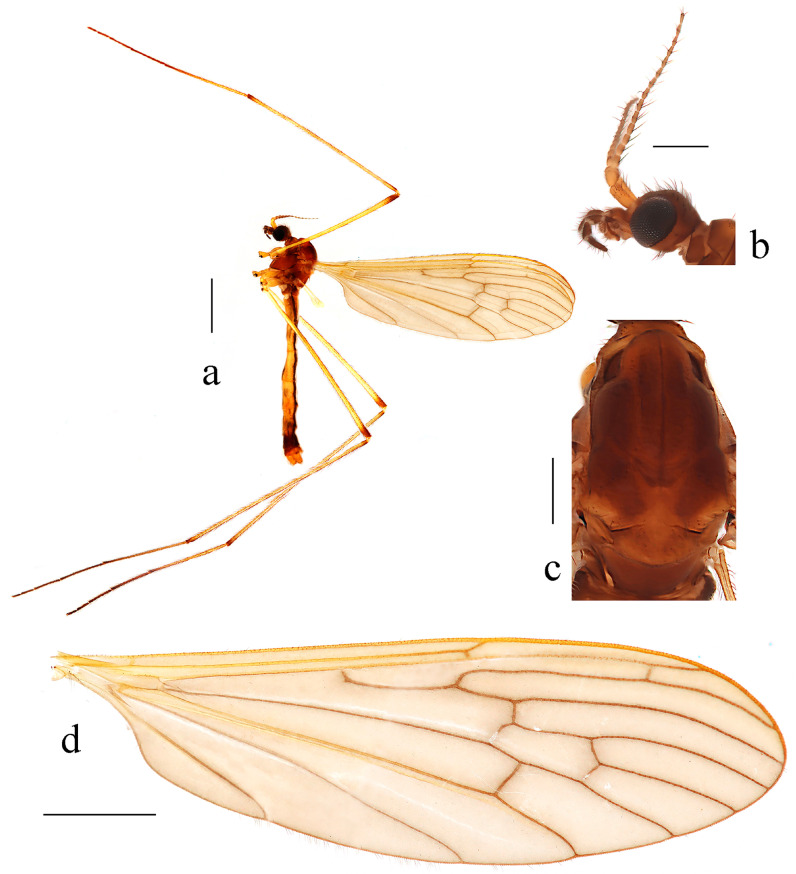
*Dicranoptycha prolongata* Alexander, 1938. (**a**) Habitus of male, lateral view; (**b**) head, lateral view; (**c**) thorax, dorsal view; (**d**) wing. Scale bars: 2.0 mm (**a**); 0.5 mm (**b**,**c**); 1.5 mm (**d**).

Head (Figure 4b), brown. Hairs on head dark brown. Antenna length 1.9–2.0 mm; scape and pedicel dark yellow, flagellomeres dark yellow at base, remaining flagellomeres brown. Scape long and cylindrical, 2.5 times as long as wide; pedicel globular, widened distally, nearly as wide as scape; flagellomeres subcylindrical, tapering apically. Setae on flagellomeres just slightly exceeding the length of respective segments. Mouthparts brown with dark brown hairs; palpus dark brown, hairs on palpus dark brown.

Thorax (Figure 4c). Prescutum and presutural scutum brown with three broad dark brown longitudinal stripes, median stripe subdivided by a narrow line of brownish black. Postsutural scutum dark brown with a brown median area. Scutellum brown. Mediotergite dark brown. Pleuron (Figure 4a) dark brown with indistinct black longitudinal stripe. Hairs on thorax white. Coxae and trochanters yellow; femora yellow with tips narrowly dark brown; tibiae yellow with tips narrowly dark brown; first tarsal segments dark yellow with tips narrowly dark brown, remaining tarsal segments dark brown. Hairs on legs brown. Wing (Figure 4d) wide, uniformly tinged with brownish yellow, except yellow costal field. Stigma indistinct, not darker than the remaining wing area. Veins brown, CuA and veins at wing base and in costal area yellow. Venation: Sc long, ending beyond fork of Rs; sc-r a short distance before the tip of Sc, a distal section of Sc twice as long as sc-r; Rs subequal to or slightly longer than cell dm; cell dm elongated, about three times as long as wide; m-cu about its length beyond fork of M, approximately at two-fifth of cell dm. Halter pale yellow, its length 1.3–1.4 mm.

Abdomen (Figure 4a). Tergite 1 brown, tergites 2–6 brownish yellow; tergite 7 brown, tergite 8 dark brown, tergite 9 brownish yellow. Sternites 1–7 brownish yellow, sternite 8 dark brown, segment 9 dark brown to brownish yellow. Hairs on abdomen white. Abdomen with conspicuous brownish black longitudinal stripes.

**Figure 5:**
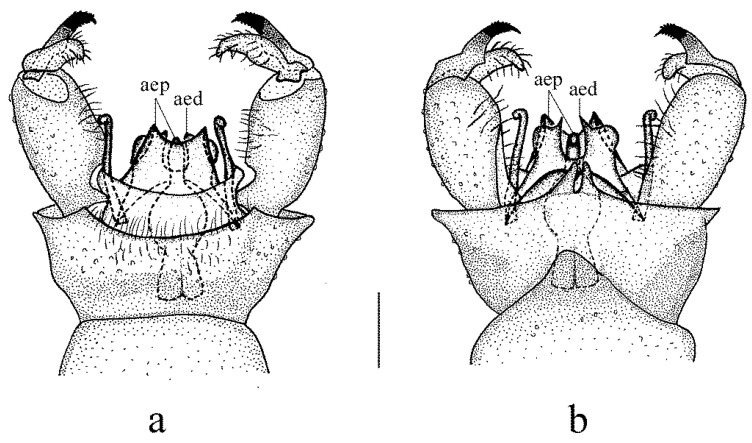
*Dicranoptycha prolongata* Alexander, 1938. (**a**) Male hypopygium, dorsal view; (**b**) male hypopygium, ventral view. Scale bar: 0.2 mm.

**Figure 6:**
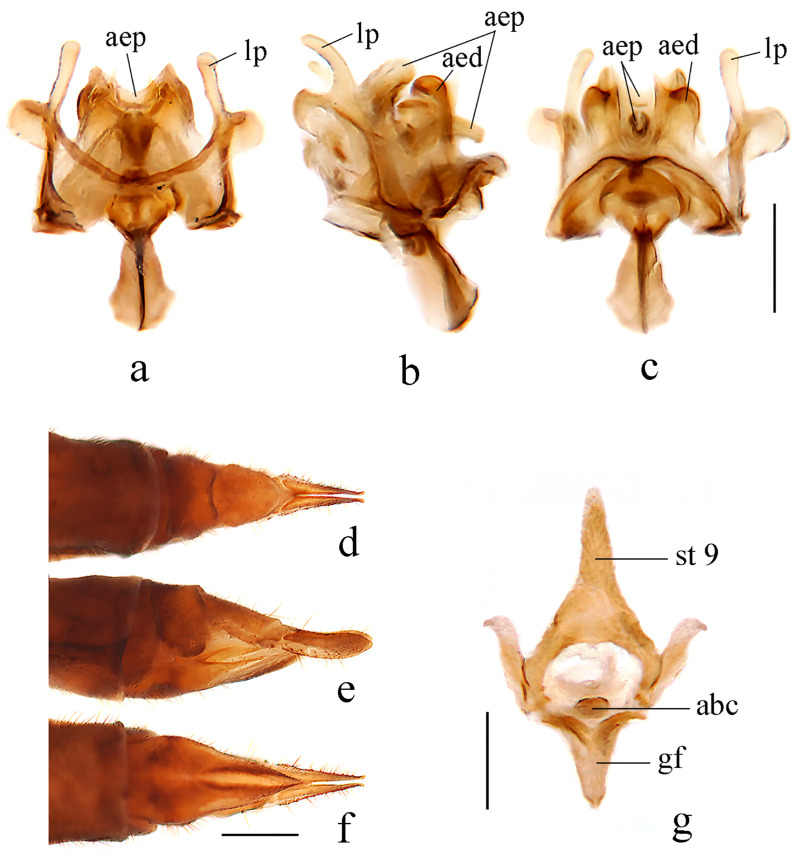
*Dicranoptycha prolongata* Alexander, 1938. (**a**) Complex of aedeagus, dorsal view; (**b**) complex of aedeagus, lateral view; (**c**) complex of aedeagus, ventral view; (**d**) female ovipositor, dorsal view; (**e**) female ovipositor, lateral view; (**f**) female ovipositor, ventral view; (**g**) vaginal apodeme. Scale bars: 0.2 mm (**a**–**c**,**g**); 0.5 mm (**d**–**f**).

Hypopygium (Figure 5 and Figure 6a–c). Posterior margin of tergite 9 with a broad and shallow round emargination. Posterior margin of sternite 9 with two long, round-tipped medial lobes and deep narrow grooves between them. Gonocoxite simple, cylindrical. Outer gonostylus sclerotized distally and curved inwards, with pointed apex, outer and inner margins of the tip serrated. Inner gonostylus nearly as long as outer gonostylus, wide and fleshy, curved at two-thirds of its length, with a blunt apex and covered with sparse setae. Lateral process slender and straight, tip blunt and strongly curved dorsally (Figure 5). Anterior apodemes fused, each slender at the point of fusion with vesica, widened at the tip. Aedeagal process with flattened upper part emarginates at the posterior margin, hook-like lower part bent downward across aedeagus. Aedeagus with a U-shaped emargination, each side with the apex pointed medially (Figure 5 and Figure 6a–c).

Female. Body length 10.0–10.5 mm, wing length 9.0–10.0 mm. Similar to male, but tergite 8 brownish yellow. Tergite 9 brownish yellow with the basal half darker, posterior margin with a broad emargination. Tergite 10 brownish yellow with posterior margin paler. Cercus brownish yellow, caudal half widened and apex blunt. Hypogynial valve reaching approximately two-thirds of the cercus, setae on dorsal margin long and distinct (Figure 6d–f). Vaginal apodeme as shown in Figure 6g, aperture of bursa copulatrix oval.

Specimens examined. 2 males, 5 females, China: Beijing Municipality, Mentougou District, Xiaolongmen National Forest Park, Xiaolongmen forest farm (39°57′53″ N, 115°25′57″ E, 1143 m), 20 September 2017, Hu Li (Malaise trap). 1 female, China: Beijing Municipality, Mentougou District, Xiaolongmen National Forest Park, Xiaolongmen forest farm (39°57′53″ N, 115°25′57″ E, 1143 m), 16 June 2017, Siyu Gong. 1 female, China: Beijing Municipality, Mentougou District, Xiaolongmen National Forest Park, scientific experimental area (1176 m), 20 September 2017, Hu Li (Malaise trap). 1 female, China: Beijing Municipality, Mentougou District, Mount Lingshan, halfway up the mountain (40°00′48″ N, 115°29′27″ E, 1206 m), 20 September 2017, Hu Li (Malaise trap).

Distribution. China (Beijing); Russia; North Korea; South Korea.

Remarks. This species was known previously from the Far East of Russia (Primorskiy Kray), North Korea, and South Korea. Now it is recorded in China for the first time. Meanwhile, the discovery of this species indicates that the tribe Dicranoptychini is recorded in Beijing, China for the first time. For descriptions and illustrations of this species, also see Alexander (1938) [70], Podenas and Byun (2014) [24], and Podenas et al. (2015) [25]. In Oosterbroek (2023), it was pointed out that the figures in Podenas and Byun (2014) may refer to an undescribed species, which is different from the figures of *D. prolongata* presented in Podenas et al. (2015) [2]. As shown in Figure 6a–c, it is more likely that the male hypopygium presented by Podenas and Byun (2014) [24] was in ventral view, whereas Podenas et al. (2015) [25] presented it in dorsal view.

#### 3.1.3. *Dicranoptycha shandongensis* Xu, Chen, and Zhang, sp. nov.

Diagnosis. Antenna with scape and pedicel brownish yellow; first flagellomere brownish yellow with brownish black tip, flagellomeres 2–5 brownish black with bases brownish yellow, remaining flagellomeres brownish black. Setae on flagellomeres just slightly exceeding the length of respective segments. Prescutum and presutural scutum brownish black. Pleuron brownish black with anepimeron and dorsal half of katepisternum brownish yellow. Wing with stigma indistinct, not darker than the remaining wing area. Rs slightly longer than cell dm; cell dm about three times as long as wide; m-cu about two-thirds of its length beyond fork of M and approximately at one-third of cell dm. Outer gonostylus with outer margin serrated. Aedeagal process arrowhead-shaped, connected beneath the margin to a rod-like lower part. Aedeagus short and broad, bifurcated, each with a hook-like sclerite at the subbase. Aperture of bursa copulatrix in females with two rod-like branches.

Description. Male. Body length 10.6–11.0 mm, wing length 8.5–8.9 mm.

**Figure 7:**
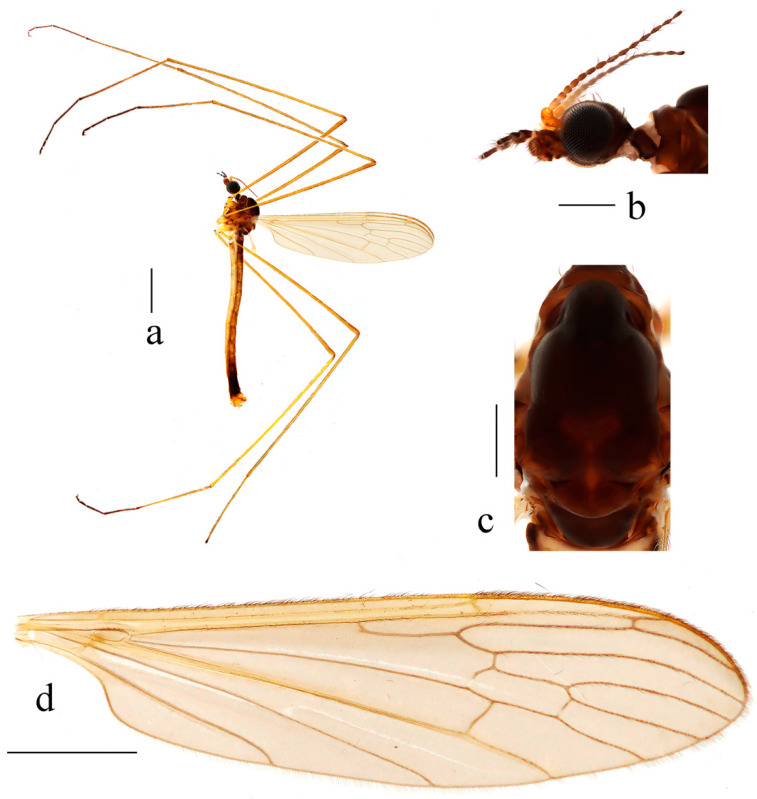
*Dicranoptycha shandongensis* sp. nov. (**a**) Habitus of male, lateral view; (**b**) head, lateral view; (**c**) thorax, dorsal view; (**d**) wing. Scale bars: 2.0 mm (**a**); 0.5 mm (**b**,**c**); 1.5 mm (**d**).

Head (Figure 7b), brownish black. Hairs on head brownish black. Antenna length 1.6–1.8 mm; scape and pedicel brownish yellow; first flagellomere brownish yellow with brownish black tip, flagellomeres 2–5 brownish black with bases brownish yellow, remaining flagellomeres brownish black. Scape long and cylindrical, twice as long as wide; pedicel globular, widened distally, slightly wider than scape; flagellomeres subcylindrical, tapering apically, first flagellomere elongated. Setae on flagellomeres just slightly exceeding the length of respective segments. Mouthparts dark brown with brownish black hairs; palpus brownish black with first segment brown; hairs on palpus brownish black.

Thorax (Figure 7c). Pronotum dark brown. Prescutum and presutural scutum brownish black. Postsutural scutum brownish black, paler medially and laterally. Scutellum brownish black, slightly paler medially and posteriorly, with anterior margin black laterally. Mediotergite brownish black. Pleuron (Figure 7a) brownish black with anepimeron and dorsal half of katepisternum brownish yellow. Hairs on thorax white. Fore coxae dark brown with paler tip, middle and hind coxae yellow; trochanters yellow; femora and tibiae dark yellow; first tarsal segments dark yellow with narrowly brownish black tips, remaining tarsal segments brownish black. Hairs on legs brownish black. Wing (Figure 7d) uniformly tinged with brownish grey, except yellowish costal field. Stigma indistinct, not darker than the remaining wing area. Veins brown, CuA and veins at wing base and in costal area brownish yellow. Venation: Sc long, ending beyond fork of Rs, sc-r close to the tip of Sc; Rs slightly longer than cell dm; cell dm elongated, about three times as long as wide; m-cu about two-thirds of its length beyond fork of M, approximately at one-third of cell dm. Halter white, its length 1.3–1.4 mm.

Abdomen (Figure 7a). Tergites 1–2 brownish black; tergites 3–6 brownish yellow; sternites 1–6 dark yellow; segments 7–8 brownish black; segment 9 dark yellow with a dorsal side brownish black. Hairs on abdomen white.

**Figure 8:**
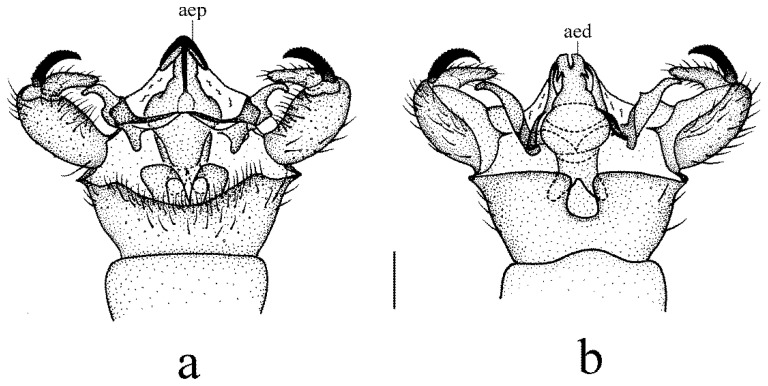
*Dicranoptycha shandongensis* sp. nov. (**a**) Male hypopygium, dorsal view; (**b**) male hypopygium, ventral view. Scale bar: 0.2 mm.

**Figure 9:**
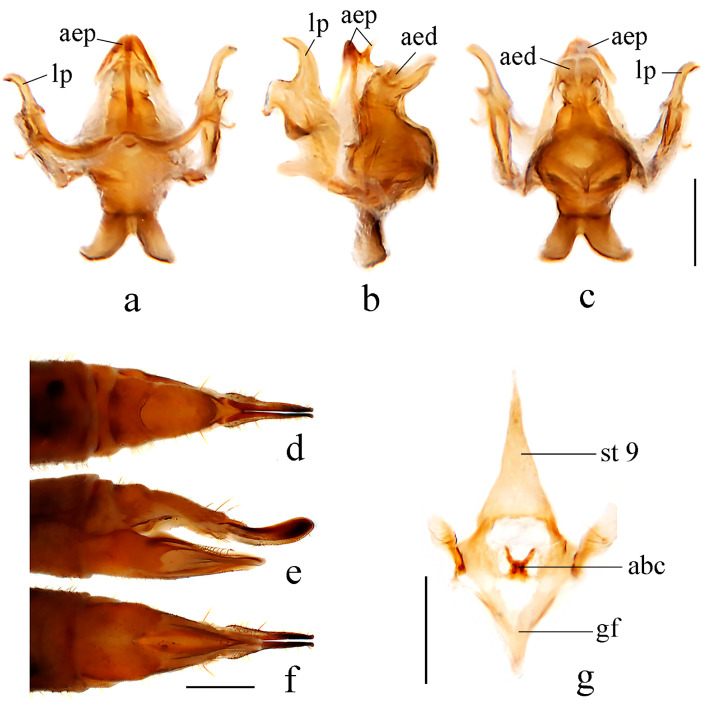
*Dicranoptycha shandongensis* sp. nov. (**a**) Complex of aedeagus, dorsal view; (**b**) complex of aedeagus, lateral view; (**c**) complex of aedeagus, ventral view; (**d**) female ovipositor, dorsal view; (**e**) female ovipositor, lateral view; (**f**) female ovipositor, ventral view; (**g**) vaginal apodeme. Scale bars: 0.2 mm (**a**–**c**,**g**); 0.5 mm (**d**–**f**).

Hypopygium (Figure 8 and Figure 9a–c). Posterior margin of tergite 9 with a broad and shallow V-shaped emargination. Posterior margin of sternite 9 with a U-shaped emargination. Gonocoxite simple, elongate-oval. Outer gonostylus sclerotized and curved inwards with pointed apex, outer margin serrated. Inner gonostylus longer than the outer gonostylus, wide and fleshy, curved at one-third of its length, with a blunt apex, and covered with sparse setae. Lateral process curved outwards, wide at the base, and narrowed distally, apex blunt (Figure 8). Anterior apodemes wide at the point of fusion with vesica, narrowed at the tip. Aedeagal process arrowhead-shaped, connected beneath the margin to a rod-like lower part. Aedeagus short and broad, bifurcated, each with a hook-like sclerite at the subbase (Figure 8 and Figure 9a–c).

Female. Body length 10.0–12.0 mm, wing length 9.0–10.0 mm. Similar to male, but abdomen with segments 5–7 dark brown. Tergite 8 brownish yellow. Tergite 9 brownish yellow with two basal brown spots, posterior margin with a broad V-shaped emargination. Tergite 10 brownish yellow with brown posterior area. Sternite 8 brownish yellow. Cercus brownish yellow with tip and dorsal part of base brownish black, caudal half widened, and apex blunt. Hypogynial valve reaching approximately the middle of cercus, setae on dorsal margin long and distinct (Figure 9d–f). Vaginal apodeme as shown in Figure 9g, aperture of bursa copulatrix with two rod-like branches.

Type material. Holotype: male, China: Shandong Province, Mengyin County, Zhongshan Temple (35°47′54″ N, 118°10′8″ E, 306 m), 23 June 2021, Yuanyuan Xu. Paratypes: 2 males 4 females, same data as holotype. 1 male, China: Shandong Province, Mengyin County, Zhongshan Conference Center (35°48′3″ N, 118°10′7″ E, 318 m), 22 June 2021, Yuanyuan Xu (light trap). 1 male, China: Shandong Province, Laoshan District, Mount Laoshan, Beijiushui, 22 June 2022, Yuanyuan Xu. 2 males and 2 females, China: Shandong Province, Laoshan District, Mount Laoshan, Beijiushui (36°12′8″ N, 120°34′54″ E, 428 m), 17 June 2019, Xiao Zhang. 1 male and 1 female, China: Shandong Province, Laoshan District, Mount Laoshan, Beijiushui (36°12′42″ N, 120°35′47″ E, 303 m), 17 June 2019, Xingyang Qian (light trap).

Distribution. China (Shandong).

Etymology. The species name *shandongensis* (adjective, feminine) refers to Shangdong, a province where the type locality is situated.

**Figure 10:**
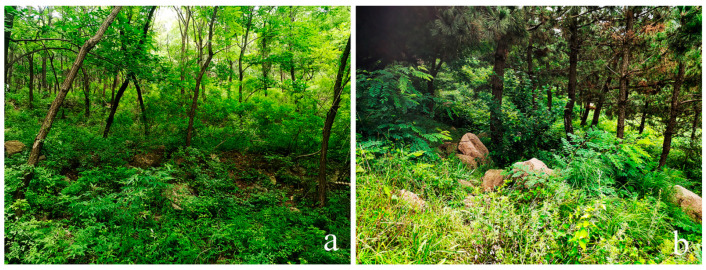
*Dicranoptycha shandongensis* sp. nov. (**a**) Habitat of Zhongshan Temple, Mengyin, Shandong, China; (**b**) habitat of Beijiushui, Mount Laoshan, Laoshan, Shandong, China.

Habitat. Adults were collected in dryer places in forests and shrubs (Figure 10). The species can be attracted by light.

Remarks. This species is somewhat similar to the *D. gyebangsaniana* presented by Podenas, Byun, and Kim, 2015, (see Figure 8 in Podenas et al. 2015 [25]) from South Korea and *D. venosa* by Alexander, 1924 (see Figure 14 in Podenas et al. 2015 [25]) from the Far East of Russia, South Korea, and Japan with a similar male hypopygium, but it can be easily distinguished from the latter two by the antenna with the scape and pedicel brownish yellow and the flagellomeres light brownish yellow at the base, turning darker (brownish black) distally (Figure 7b), the uniform brownish black prescutum and presutural scutum (Figure 7c), and dark yellow femora with the tips not darker or lighter than the remaining parts (Figure 7a). In *D. gyebangsaniana*, the flagellomeres are light reddish brown at the base and turn lighter (yellow) towards the distal end, the prescutum and presutural scutum are brown with indistinct longitudinal stripes, and the femora are obscure yellow and become dark brown in the distal third [25]. In *D. venosa*, the antenna has a black scape and obscure yellow pedicel (that is, the pedicel is conspicuously lighter than the scape), the prescutum and presutural scutum have four indistinct dark brown stripes, the fore femur is black with the basal third yellow and other femora obscure yellow with the basal third or shorter basal part black [71]. This species is also somewhat similar to *D. fuscescens* by Schummel, 1829, widely distributed in the Palaearctic region with a similar wing *venation* (see the only figure in Stubbs & Little 1974 [17]) and the male hypopygium (see Figure 3 in Starý 1972 [22]), but it can be distinguished from the latter by the uniformly brownish black prescutum and presutural scutum (Figure 7c), the dark yellow legs with tarsi brownish black (Figure 7a), and aperture of bursa copulatrix of female ovipositor with two rod-like branches (Figure 9g). In *D. fuscescens*, the prescutum and presutural scutum have dark longitudinal stripes, the legs are uniformly testaceous to brown with only the bases lighter, and the aperture of bursa copulatrix has no branches [22]. The discovery of this species indicates that the tribe Dicranoptychini is recorded in Shandong, China for the first time.

#### 3.1.4. Key to the Chinese Species of the Genus Dicranoptycha

1. Rs nearly as long as cell dm, no more than 1.2 times the length of cell dm (Figure 1d, Figure 4d and Figure 7d)…………………………………………………………………………………2

- Rs distinctly longer than cell dm, at least 1.5 times the length of cell dm……………8

2. Wing with stigma and outer costal region dark………*D. geniculata* Alexander, 1928

- Wing with stigma and outer costal region not darker than the remaining wing area (Figure 1d, Figure 4d and Figure 7d)……………………………………………………………………3

3. Pleuron with conspicuous darker or lighter markings (Figure 4a and Figure 7a)…………4

- Pleuron without markings (Figure 1a)……………………………………………………6

4. Prescutum and presutural scutum uniformly brownish black (Figure 7c)…………………………………………………………………*D. shandongensis* sp. nov.

- Prescutum and presutural scutum with three stripes (Figure 4c)……………………5

5. Aedeagal process with outer portion paired and lyriform, each arm obtuse and deeply split at apex……………………………………*D. phallosomica* Alexander, 1937

- Aedeagal process with flattened upper part emarginate at posterior margin, hook-like lower part bent downward across aedeagus (Figure 5 and Figure 6a–c)…………………………………………………………*D. prolongata* Alexander, 1938

6. Prescutum and presutural scutum with longitudinal stripes according to Alexander (1928, 1931) [72,73] (Figure 4c)…………………………*D. pallidibasis* Alexander, 1931

- Prescutum and presutural scutum without markings (Figure 1c and Figure 7c)……………7

7. Legs with femora yellow, a little brighter in basal portion, according to Alexander (1935) [74]………………………………………………………*D. vulpes* Alexander, 1935

- Legs with femora brownish yellow with dark brown tip (Figure 1a)…………………………………………………………………*D. jiufengshana* sp. nov.

8. Femora of the legs black with one-fourth of the basal obscure yellow…………………………………………………………*D. nigrotibialis* Alexander, 1934

- Femora of the legs uniformly yellow or only the tips narrowly darker (Figure 4a and Figure 7a)………………………………………………………………………………………9

9. Wing with stigma elongated, dark brown; pleuron uniformly grey, according to Alexander (1930) [75]……………………………………………*D. issikina* Alexander, 1930

- Wing with stigma indistinct, not darker than the remaining wing area (Figure 1d, Figure 4d and Figure 7d); pleuron with conspicuous darker or lighter markings (Figure 4a and Figure 7a)…………………………………………………………………………………………10

10. Abdomen with the first tergite dark brown, remaining tergites obscure yellow and dark brown laterally, according to Alexander (1928) [76]; inner gonostylus approximately 1.5 times the length of outer gonostylus…………………………………………………………*D. formosensis* Alexander, 1928

- Abdomen with tergites uniformly dark brown or brownish yellow according to Alexander (1942) [77]; inner gonostylus approximately as long as outer gonostylus……………………………………………………*D. kwangtungensis* Alexander, 1942

### 3.2. Mitochondrial Genome of D. shandongensis *sp. nov.*

**Figure 11:**
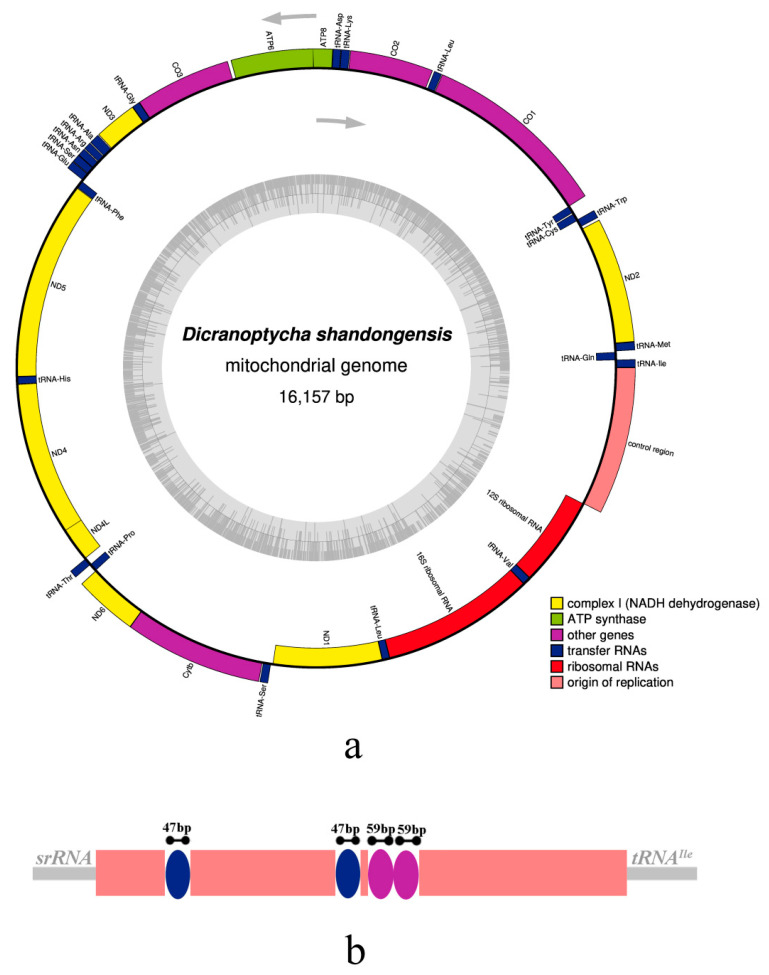
Gene map (**a**) and control region (**b**) of the mitochondrial genome of *Dicranoptycha shandongensis* sp. nov.

In this study, we sequenced and annotated the complete mt genome of *D. shandongensis* (GenBank accession no. OR074105), which represents the first mt genome of the crane fly tribe Dicranoptychini. The mt genome is 16,157 bp in length and its gene composition and arrangement are identical to the presumed ancestral insect mt genomes [78] (Figure 11a). Thirteen protein-coding genes (PCGs), twenty-two transfer RNA (tRNA) genes, two ribosomal RNA (rRNA) genes, and a control region (AT-rich region) are detected. For PCGs, *COI*, *COII*, *COIII*, *CytB*, *ATP6*, *ATP8*, *ND2*, *ND3*, and *ND6* are coded in the majority strand, whereas *ND4*, *ND4L*, *ND5*, and *ND1* are coded in the minority strand (Figure 11a). The lengths of tRNA genes range from 63 bp to 72 bp, whereas the lengths of large rRNA (lrRNA) and small rRNA (srRNA) genes are 1331 bp and 788 bp, respectively (Table S1). The control region is 1212 bp in length and located between the srRNA gene and *tRNA^Ile^*, including a total of 16 (TA)n regions, of which 10 are (TA)3, 3 are (TA)4, 1 is (TA)5, 1 is (TA)6, and 1 is (TA)7. In addition, there are two tandem repeat elements with a length of 59 bp and two 47 bp repeat elements with an interval of 329 bp in the control region (Figure 11b). The organization of the mt genome is generally compact. The intergenic spacers other than the control region are 19 in number and generally less than 26 bp, and the maximum intergenic spacer exists between *ATP6* and *COIII*. The mt genome also has overlapping genes and most neighboring genes overlap by no more than 8 bp.

**Figure 12:**
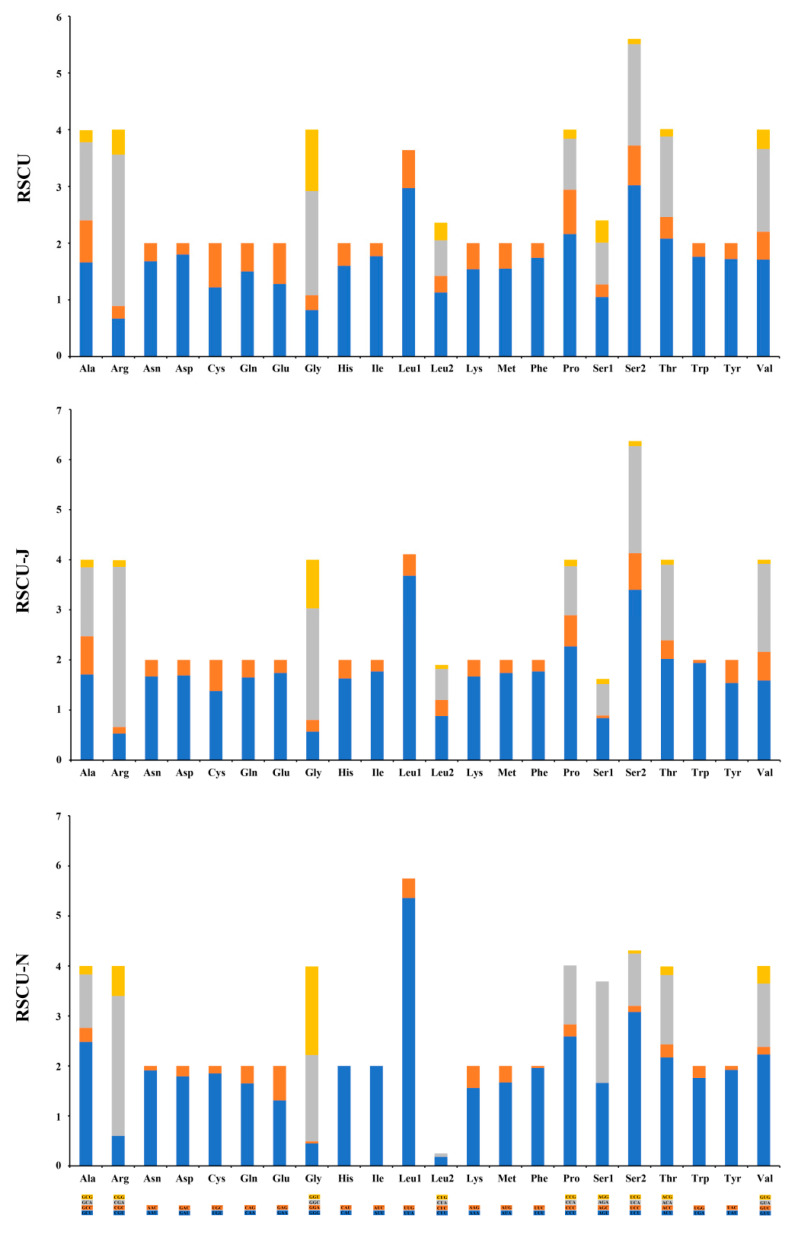
Relative synonymous codon usage (RSCU) of the protein-coding genes in the mitochondrial genome of *Dicranoptycha shandongensis* sp. nov. Leu1 = Leu (UUR); Leu2 = Leu (CUN); Ser1 = Ser (AGN); Ser2 = Ser (UCN).

All PCGs of the mt genome show the typical ATN start codons (ATT/ATG) and the typical TAR (TAA/TAG) stop codons, except that TCG is the start codon for *COI* (Table S1). The 62 codons of PCGs, with a total number of 3739, determine 22 amino acids, of which Leu (UUR) and Phe are the 2 most frequently used amino acids, and Arg and Cys are the least used amino acids (Table S2). Relative synonymous codon usage (RSCU) is also provided (Figure 12), which reflects how often a particular codon is used relative to the expected number of times that codon would be used in the absence of codon usage bias.

The whole mt genome and its five major genome partitions (i.e., PCGs, tRNA genes, lrRNA gene, srRNA gene, and the control region) are biased to a high content of AT bases (77.1%, 74.3%, 78.1%, 81.3%, 79.2%, and 92.9%) (Table 3). The AT content of the control region is the highest, which is consistent with the trend of mt genomes in other crane flies [5]. This species shows positive AT-skew for the whole mt genome, tRNA genes, and the control region (0.00, 0.02, and 0.04); negative AT-skew for PCGs, lrRNA, and srRNA (−0.17, −0.05, and −0.01); negative GC-skew for the whole mt genome and the control region (−0.19 and −0.30); and positive GC-skew for PCGs, tRNA genes, lrRNA, and srRNA (0.04, 0.14, 0.32, and 0.22) (Table 3). For this species, the whole mt genome and the five major partitions all had the same trends in the AT content, AT, and GC-skews, which display similar patterns and are consistent with the common base composition biases of mt genomes of Tipuloidea [5].

### 3.3. Phylogenetic Analyses

**Figure 13:**
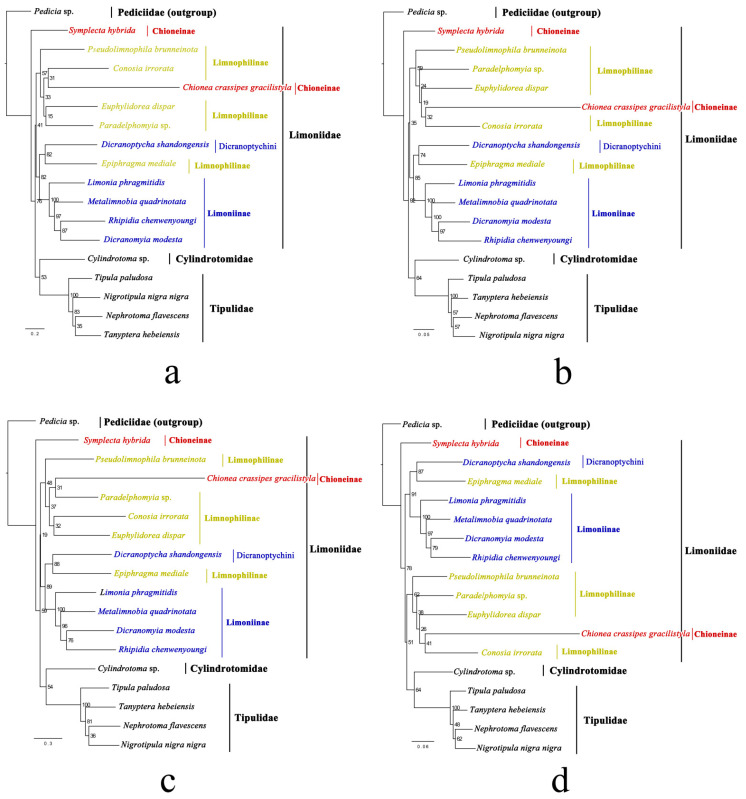
Phylogenetic trees of Tipuloidea inferred from the datasets (**a**) PCGRNA, (**b**) PCG12RNA, (**c**) PCG, and (**d**) PCG12 under the Bayesian inference (BI) method. The number at each node is the posterior probability (PP).

**Figure 14:**
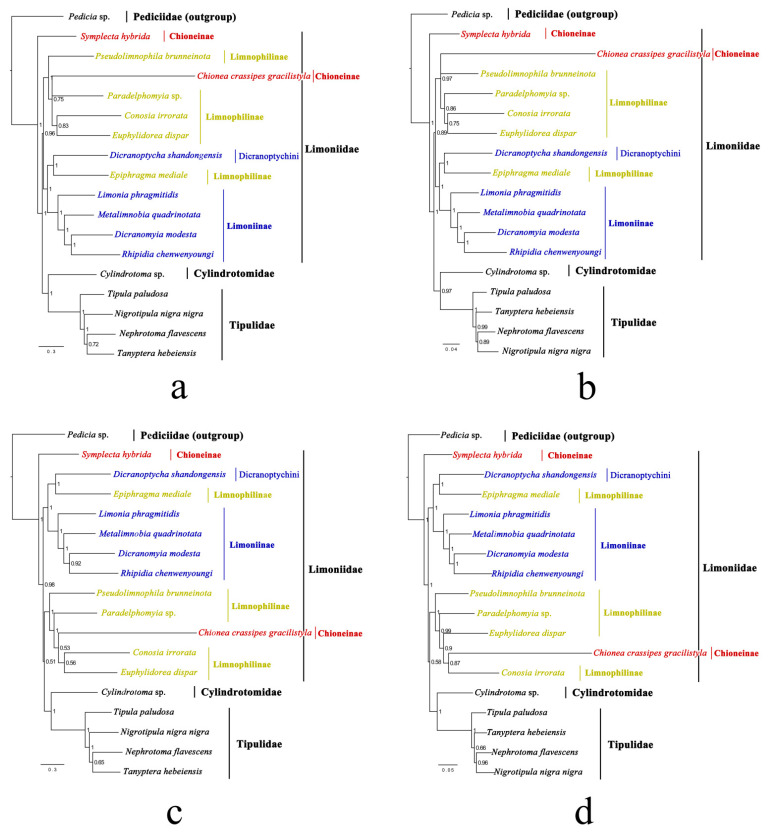
Phylogenetic trees of Tipuloidea inferred from the datasets (**a**) PCGRNA, (**b**) PCG12RNA, (**c**) PCG, and (**d**) PCG12 under the maximum likelihood (ML) method. The number at each node is the bootstrap value (BV).

Eighteen representatives from all four families of Tipuloidea were included in the phylogenetic analysis. Eight phylogenetic trees inferred from the four datasets under BI and ML methods were constructed (Figure 13 and Figure 14).

In all BI and ML trees, *Symplecta* (Chioneinae) is a sister taxon to all non-pediciid crane flies (100% or 98% PP for all BI trees; 76%, 92%, 59%, and 78% BVs for ML trees). The monophyly of the remaining Limoniidae is supported in the trees inferred from the PCGRNA and PCG12RNA datasets under the BI method (96% and 89% PPs), as well as in the trees inferred from the PCG, PCGRNA, and PCG12RNA datasets under the ML method with low BVs (41%, 35%, and 19%). In the trees inferred from the PCG and PCG12 datasets under the BI method and the PCG12 dataset under the ML method, there is also a weakly supported arrangement; that is, the Limnophilinae and *Chionea* (Chioneinae) are a sister group to a clade of Cylindrotomidae and Tipulidae (51% and 58% PPs, 51% BV). However, Limoniidae is not supported as a monophyletic clade in all phylogenetic trees. The monophyly of Tipulidae is strongly supported (100% PP/BV for all trees). The sister relationship between Cylindrotomidae and Tipulidae is supported (100% or 97% PP for all BI trees; 53%, 64%, 54%, and 64% BVs for ML trees), which was accepted by Oosterbroek and Theowald (1991) [11], Ribeiro (2008) [4], Petersen et al. (2010) [3], Zhang et al. (2016) [5], and Kang et al. (2017) [6].

Our Limoniidae species (excluding *Symplecta*) are strongly supported to be divided into two well-supported clades: a clade of the Limoniinae and *Epiphragma* (Limnophilinae) (100% PP for all BI trees; 82%, 85%, 89% and 91% BVs for ML trees) and a clade of the Limnophilinae and *Chionea* (100% or 97% PP for all BI trees; 57%, 59%, 48%, and 62% BVs for ML trees). Therefore, in this study, the monophyly of both Limoniinae and Limnophilinae with their current composition of genera is not supported, which was also proposed by Oosterbroek and Theowald (1991) based on 105 morphological characters of larvae and pupae [11], by Ribeiro (2008) based on 88 morphological characters [4], and by Petersen et al. (2010) based on the combined morphological characters and two nuclear genes [3]. However, it does not mean that the Limoniinae and Limnophilinae are artificial groups, and it is more likely that the position of some genera in the classification of Limoniidae is erroneous. Due to insufficient sampling and the problem of the long branch attraction, further studies with more representatives are needed to confidently define the monophyly of the Chioneinae.

Within the clade of Limoniinae and *Epiphragma*, the sister relationship between Dicranoptychini and *Epiphragma* (100% PP for all BI trees; 82%, 74%, 88%, and 87% BVs for ML trees) and the monophyly of the remaining Limoniinae (100% PP/BV for all trees) are strongly supported in all phylogenetic trees. This means that the position of *Epiphragma* in the classification of Limoniidae may be erroneous. Savchenko (1986) mentioned that *Epiphragma* differs significantly from all other genera of the Limnophilinae in several important characters [79]. This genus should possibly be transferred to Limoniinae, or it may be a possible transition to Limoniinae. Dicranoptychini is more likely to represent a basal lineage within the Limoniinae.

## 4. Conclusions

The taxonomic study here is intended to improve the knowledge of Dicranoptychini in China. Two new species and a newly recorded species from China are found in this study, which increases the number of Dicranoptychini species in the Chinese Mainland from four to seven, and adds three new provincial distribution records for the tribe in China. This study also presented the first mt genome of the tribe, which is a typical circular DNA molecule with a length of 16,157 bp. The newly sequenced mt genome shows a similar gene order, nucleotide composition, and codon usage to mt genomes of other crane flies. It is worth noting that two tandem repeat elements with a length of 59 bp, and two 47 bp repeat elements with an interval of 329 bp are found in the control region. Phylogenetic analysis indicates that the position of the genus *Epiphragma* in the classification of Limoniidae needs to be revised, and Dicranoptychini may be a basal lineage within the Limoniinae. This study also reconfirms the traditional view that Cylindrotomidae and Tipulidae have a sister-group relationship. The phylogenetic results in this study are very preliminary, and a more complex history of independent lineages of Limoniidae needs to be revealed based on more data in the future.

## Figures and Tables

**Table 1:** Information on the types of Chinese *Dicranoptycha* species examined in this study.

Species	Specimens Examined	Collection
*D. formosensis*	Holotype, male, China: Taiwan, Ranrun (333 m), 2 June 1927, S. Issiki.	USNM
*D. geniculata*	Paratype, male, China: Taiwan (without exact locality data), October 1926, S. Issiki.	USNM
*D. issikina*	Holotype, male, China: Taiwan, Hassensan (1167–1833 m), 22 October 1929, S. Issiki. Paratype, male, same collection data as holotype.	USNM
*D. kwangtungensis*	Allotype, male, China: Guangdong, Lin District, Lung Ping Hui, 16–17 May 1934, F. K. To.	USNM
*D. nigrotibialis*	Holotype, female, China: Taiwan, Fudieda (1567 m), 13 August 1933, S. Issiki.	USNM
*D. pallidibasis*	Allotype, female, Japan: Honshiu, Norikuradake, Japanese Alps, Shinano, 26 July 1929, J. Machida.	USNM
*D. phallosomica*	Holotype, male, China: Jiangxi, Kuling (1080 m), 15 July 1935, O. Piel.	IOZ
	Paratype, male, China: Jiangxi, Hong San (1000 m), 24 June 1936, J. L. Gressitt.	USNM
*D. vulpes*	Paratype, female, China: Sichuan, Beh Luh Din (30 miles north of Chengdu, 2000 m), 18–25 August 1933, J. L. Graham.	USNM

**Table 3:** Nucleotide composition of the mitochondrial genome of *Dicranoptycha shandongensis* sp. nov.

Feature	A%	T%	C%	G%	A + T%	C + G%	AT-Skew	GC-Skew
Whole mitgenome	38.6	38.5	13.6	9.3	77.1	22.9	0.00	−0.19
PCGs	30.9	43.4	12.4	13.3	74.3	25.7	−0.17	0.04
tRNA genes	39.9	38.2	9.6	12.7	78.1	22.3	0.02	0.14
lrRNA	39.0	42.8	6.2	12.0	81.8	18.2	−0.05	0.32
srRNA	39.3	39.8	8.1	12.7	79.1	21.8	−0.01	0.22
AT-rich region	48.2	44.7	4.6	2.5	92.9	7.1	0.04	−0.30

## Data Availability

The mitochondrial sequence data sequenced in this study are openly available in the GenBank of NCBI under accession no. OR074105.

## Supplementary Materials

The following supporting information can be downloaded at: https://www.mdpi.com/article/10.3390/insects14060535/s1, Table S1: Organization of the mitochondrial genome of *Dicranoptycha shandongensis* sp. nov.; Table S2: Codon usage of the mitochondrial genome of *Dicranoptycha shandongensis* sp. nov.
