# Supplementary material for: Contribution to the Knowledge of Dicranoptychini (Diptera, Tipuloidea, Limoniidae) in China, with the First Mitochondrial Genome of the Tribe and Its Phylogenetic Implications
Source: Insects. 2023 Jun 7;14(6):535. doi: 10.3390/insects14060535 (PMC10299592; doi:10.3390/insects14060535)
Supplement: Supplementary file 1 [file insects-14-00535-s001.zip › insects-2392052-supplementary.pdf]

Table S1. Organization of the mitochondrial genome of *Dicranoptycha shandongensis* sp. nov.

Gene	Direction	Location	Size (bp)	Anticodon	Codon		Intergenic nucleotide*
					Start	Stop	
$tRNA^{Ile}$	J	1-67	67	GAT			
$tRNA^{Gln}$	N	65-133	69	TTG			-3
$tRNA^{Met}$	J	142-209	68	CAT			8
ND2	J	210-1238	1029		ATT	TAA	0
$tRNA^{Trp}$	J	1259-1326	68	TCA			20
$tRNA^{Cys}$	N	1319-1387	69	GCA			-8
$tRNA^{Tyr}$	N	1398-1462	65	GTA			10
COI	J	1461-2996	1536		TCG	TAA	-2
$tRNA^{Leu(UUR)}$	J	3001-3065	65	TAA			4
COII	J	3080-3763	684		ATG	TAA	14
$tRNA^{Lys}$	J	3768-3838	71	CTT			4
$tRNA^{Asp}$	J	3840-3905	66	GTC			1
ATP8	J	3906-4067	162		ATT	TAA	0
ATP6	J	4061-4738	678		ATG	TAA	-7
COIII	J	4765-5553	789		ATG	TAA	26
$tRNA^{Gly}$	J	5554-5618	65	TCC			0
ND3	J	5619-5972	354		ATT	TAA	0
$tRNA^{Ala}$	J	5979-6043	65	TGC			6
$tRNA^{Arg}$	J	6069-6131	63	TCG			25
$tRNA^{Asn}$	J	6137-6202	66	GTT			5
$tRNA^{Ser(AGN)}$	J	6203-6269	67	GCT			0
$tRNA^{Glu}$	J	6272-6339	68	TTC			2
$tRNA^{Phe}$	N	6352-6418	67	GAA			12
ND5	N	6422-8155	1734		ATG	TAA	3
$tRNA^{His}$	N	8156-8220	65	GTG			0
ND4	N	8222-9562	1341		ATG	TAA	1
ND4L	N	9556-9852	297		ATG	TAA	-7
$tRNA^{Thr}$	J	9855-9920	66	TGT			2
$tRNA^{Pro}$	N	9921-9988	68	TGG			0
ND6	J	9991-10515	525		ATC	TAA	2
CytB	J	10515-11651	1137		ATG	TAA	-1
$tRNA^{Ser(UCN)}$	J	11659-11725	67	TGA			7
ND1	N	11742-12689	948		ATG	TAG	16
$tRNA^{Leu(CUN)}$	N	12690-12754	65	TAG			0
lrRNA	N	12755-14085	1331				0
$tRNA^{Val}$	N	14086-14157	72	TAC			0
srRNA	N	14158-14945	788				0
AT rich region	J	14946-16157	1212				0

* Intergenic nucleotide: minus indicates overlapping between genes.

Table S2. Codon usage of the mitochondrial genome of *Dicranoptycha shandongensis* sp. nov.

Amino acid	Codon	N	RSCU	N+	RSCU	N-	RSCU
Phe(F)	**UUU(F)**	325	1.74	189	1.77	136	1.96
	UUC(F)	49	0.26	25	0.23	3	0.04
Leu^{UUR} (L)	**UUA(L)**	329	2.97	233	3.68	234	5.36
	UUG(L)	74	0.67	27	0.43	17	0.39
Leu^{CUN} (L)	**CUU(L)**	125	1.13	56	0.88	8	0.18
	CUC(L)	32	0.29	20	0.32	0	0
	CUA(L)	70	0.63	39	0.62	3	0.07
	CUG(L)	34	0.31	5	0.08	0	0
Ile (I)	**AUU(I)**	259	1.77	181	1.77	115	2
	AUC(I)	33	0.23	23	0.23	0	0
Met (M)	**AUA(M)**	158	1.55	107	1.74	92	1.67
	AUG(M)	46	0.45	16	0.26	18	0.33
Val (V)	**GUU(V)**	70	1.71	39	1.59	44	2.23
	GUC(V)	20	0.49	14	0.57	3	0.15
	GUA(V)	60	1.46	43	1.76	25	1.27
	GUG(V)	14	0.34	2	0.08	7	0.35
Ser^{UCN} (S)	**UCU(S)**	69	3.02	65	3.4	50	3.08
	UCC(S)	16	0.7	14	0.73	2	0.12
	UCA(S)	41	1.79	41	2.14	17	1.05
	UCG(S)	2	0.09	2	0.1	1	0.06
Pro (P)	**CCU(P)**	55	2.16	51	2.27	22	2.59
	CCC(P)	20	0.78	14	0.62	2	0.24
	CCA(P)	23	0.9	22	0.98	10	1.18
	CCG(P)	4	0.16	3	0.13	0	0
Thr (T)	**ACU(T)**	66	2.08	60	2.02	25	2.17
	ACC(T)	12	0.38	11	0.37	3	0.26
	ACA(T)	45	1.42	45	1.51	16	1.39
	ACG(T)	4	0.13	3	0.1	2	0.17
Ala (A)	**GCU(A)**	47	1.66	47	1.71	44	2.48
	GCC(A)	21	0.74	21	0.76	5	0.28
	GCA(A)	39	1.38	38	1.38	19	1.07
	GCG(A)	6	0.21	4	0.15	3	0.17
Tyr (Y)	**UAU(Y)**	232	1.72	86	1.54	76	1.92
	UAC(Y)	38	0.28	26	0.46	3	0.08
Stop (*)	**UAA(*)**	173	1.27	52	1.37	3	1.5
	UAG(*)	99	0.73	24	0.63	1	0.5
His (H)	**CAU(H)**	68	1.6	53	1.63	15	2
	CAC(H)	17	0.4	12	0.37	0	0
Gln (Q)	**CAA(Q)**	83	1.5	57	1.65	19	1.65
	CAG(Q)	28	0.5	12	0.35	4	0.35
Asn (N)	**AAU(N)**	117	1.68	90	1.67	61	1.91

	AAC(N)	22	0.32	18	0.33	3	0.09
Lys (K)	**AAA(K)**	76	1.54	45	1.67	32	1.56
	AAG(K)	23	0.46	9	0.33	9	0.44
Asp (D)	**GAU(D)**	90	1.8	44	1.69	17	1.79
	GAC(D)	10	0.2	8	0.31	2	0.21
Glu (E)	**GAA(E)**	74	1.28	40	1.74	21	1.31
	GAG(E)	42	0.72	6	0.26	11	0.69
Cys (C)	**UGU(C)**	22	1.22	9	1.38	25	1.85
	UGC(C)	14	0.78	4	0.62	2	0.15
Trp (W)	**UGA(W)**	72	1.76	61	1.94	29	1.76
	UGG(W)	10	0.24	2	0.06	4	0.24
Arg (R)	CGU(R)	6	0.67	4	0.53	3	0.6
	CGC(R)	2	0.22	1	0.13	0	0
	**CGA(R)**	24	2.67	24	3.2	14	2.8
	CGG(R)	4	0.44	1	0.13	3	0.6
Ser^{AGN} (S)	**AGU(S)**	24	1.05	16	0.84	27	1.66
	AGC(S)	5	0.22	1	0.05	0	0
	AGA(S)	17	0.74	12	0.63	33	2.03
	AGG(S)	9	0.39	2	0.1	0	0
Gly (G)	GGU(G)	35	0.82	17	0.57	11	0.45
	GGC(G)	11	0.26	7	0.23	1	0.04
	**GGA(G)**	78	1.84	67	2.23	42	1.73
	GGG(G)	46	1.08	29	0.97	43	1.77

N is the total number of codons used for protein coding genes. $N^+$and N^- are the codon usage numbers of protein coding genes for majority strand and minority strand, respectively. RSCU is the relative synonymous codon usage. The bold font highlights the codon with the highest frequency of use of each amino acid. The underlined font highlights codons that full match the anti codons of the tRNAs.
